# Human interaction with a virtual assistant in preparation for in-hospital orthopedic consultation. A feasibility and acceptability study in older adults with osteoarthritis

**DOI:** 10.1016/j.pecinn.2025.100446

**Published:** 2025-11-19

**Authors:** Walter van der Weegen, Thomas Timmers, Maud Jacobs, Katja Saris, Sebastiaan A.W. van de Groes

**Affiliations:** a Sports & Orthopedics Research Centre, St. Anna Hospital, Geldrop, the Netherlands; b Department of Anesthesiology, Pain and Palliative Medicine, Radboud University Medical Center, Nijmegen, the Netherlands; c IQ Health, Radboud University Medical Center, Nijmegen, the Netherlands; d Digital Care Research Department, Interactive Studios, the Netherlands; e Department of Orthopaedic Surgery, Radboud University Medical Center, Nijmegen, the Netherlands

**Keywords:** History taking, Orthopedics, Avatar, Feasibility, Acceptability

## Abstract

**Objective:**

To assess the feasibility and acceptability of an avatar-based history taking tool for patients referred for knee or hip osteoarthritis.

**Methods:**

In a single Centre study patients referred for knee or hip osteoarthritis were asked to use an avatar-based history taking tool. The technical basis of this tool was formed by speech recognition (Google Dialogflow), a “rule-based” model and ChatGPT 3.5. To assess feasibility and acceptability patients filled in a modified version of the Artificial-Social-Agent Questionnaire afterwards, with supplemental open questions.

**Results:**

Of the 40 participating patients (median age 67 years, min: 47.3, max 87.5, 26 women), 36 (87.5 %) scored the digital conversation as natural, 33 (82.5 %) found the avatar easy to use, 31 (77.5 %) were open to using the avatar more often in the future, and 26 (65 %) found the conversation captivating.

**Conclusion:**

Avatar-based history taking is feasible and accepted by older patients seeking orthopedic care for knee or hip osteoarthritis.

Innovation:

Avatar-based history taking applications could be offered to patients who are referred for knee or hip osteoarthritis, before they have their face-to-face consultation with an orthopedic surgeon.

## Highlights

• Patient-avatar hip or knee anamnesis feels natural from a patient perspective.
• Older patients find an avatar easy to use for hip or knee anamnesis.
• Older patients are willing towards future avatar use in orthopedics.
• Avatar anamnesis might support face-to-face consultation.

## 1 Introduction

In the Western world, an aging population is increasing the demand for healthcare while simultaneously reducing the workforce available to provide these services [1]. Osteoarthritis (OA), which most frequently occurs after age 40 years and prevalence increases steeply with age, is also projected to grow substantially in the next decades, thereby negatively impacting health care accessibility and availability [2].

Artificial Intelligence (AI)-based technologies are viewed as a potential solution to this challenge. However, aside from radiology, AI has not yet been fully integrated into most health care practices. One such underutilized innovation is Computer Assisted History Taking (CAHT) systems, software applications that allow patients to electronically record their medical history before face-to-face consultation with a clinician [3]. CAHT enhances the accuracy and comprehensiveness of patient history, empowers patients to play an active role in their care, and potentially reduces consultation time, keeping health care accessible [4]. The integration of AI through digital conversational agents, using Natural Language Processing (NLP) to interact with patients via speech, offers a promising approach to overcome the limitations of traditional digital questionnaires used in CAHT. In theory, conversational agents such as avatars that mimic human behavior could potentially become a Virtual Assistant (VA) which saves the clinician time while maintaining a high standard of care. However, apart from technical barriers patients might dislike to engage with human-like virtual assistants, making them feel uncomfortable and not at ease [5,6]. Although very few studies have focused on the effect of age in relation to interaction with virtual agents, there is evidence that older people respond well to health related interactions with virtual agents [7,8]. The aim of our study was therefore to further explore the feasibility and acceptability of communication with a VA, specifically designed for patients with hip or knee OA seeking consultation with an orthopedic surgeon.

## 2 Methods

### 2.1 Design

This feasibility and acceptability study was conducted in a single hospital (Anna hospital, Geldrop, The Netherlands) between May 6th 2024 and June 11th 2024.

### 2.2 Sample and setting

Patients were eligible if they were diagnosed with hip or knee osteoarthritis (OA), >18 years of age, Dutch speaking and willing to participate. Enrollment was determined a priori as a convenience sample with a minimum of 30 and a maximum of 40 patients, which is in line with comparable studies [9,10]. Patients were invited to participate after their face-to-face consultation with an orthopedic surgeon. Patients were informed that the VA was a first prototype and that the aim of the study was to explore feasibility and acceptability.

### 2.3 Intervention

**Fig. 1:**
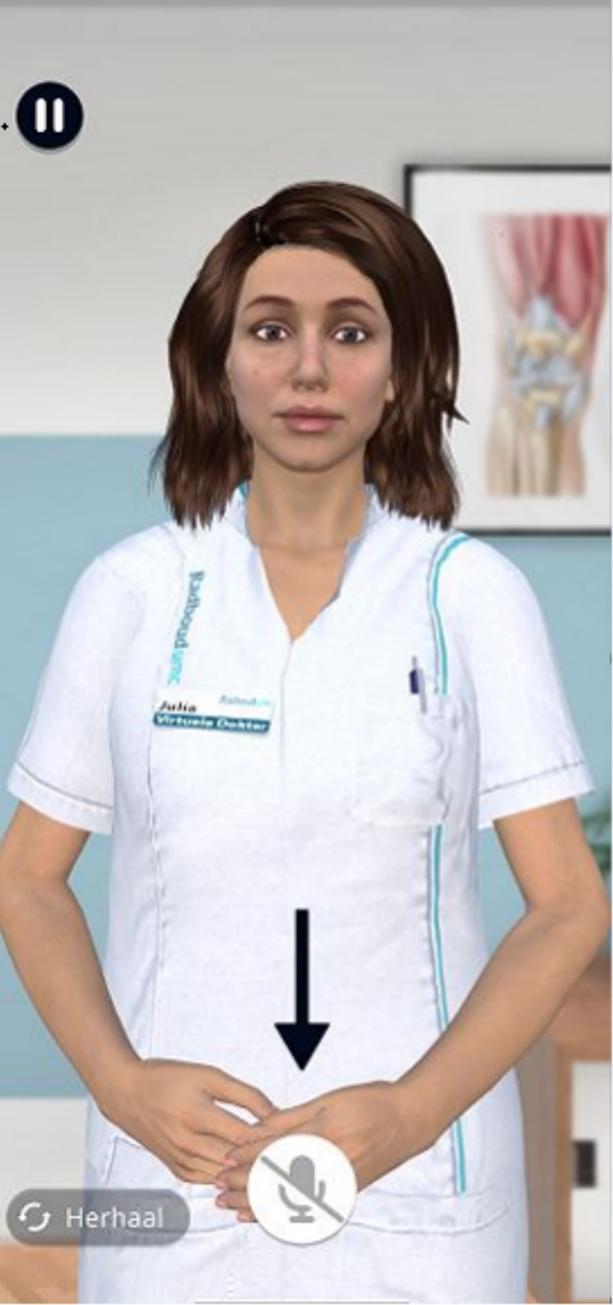
Screenshot of Julia, the virtual assistant, as it appears on a mobile phone

**Fig. 2:**
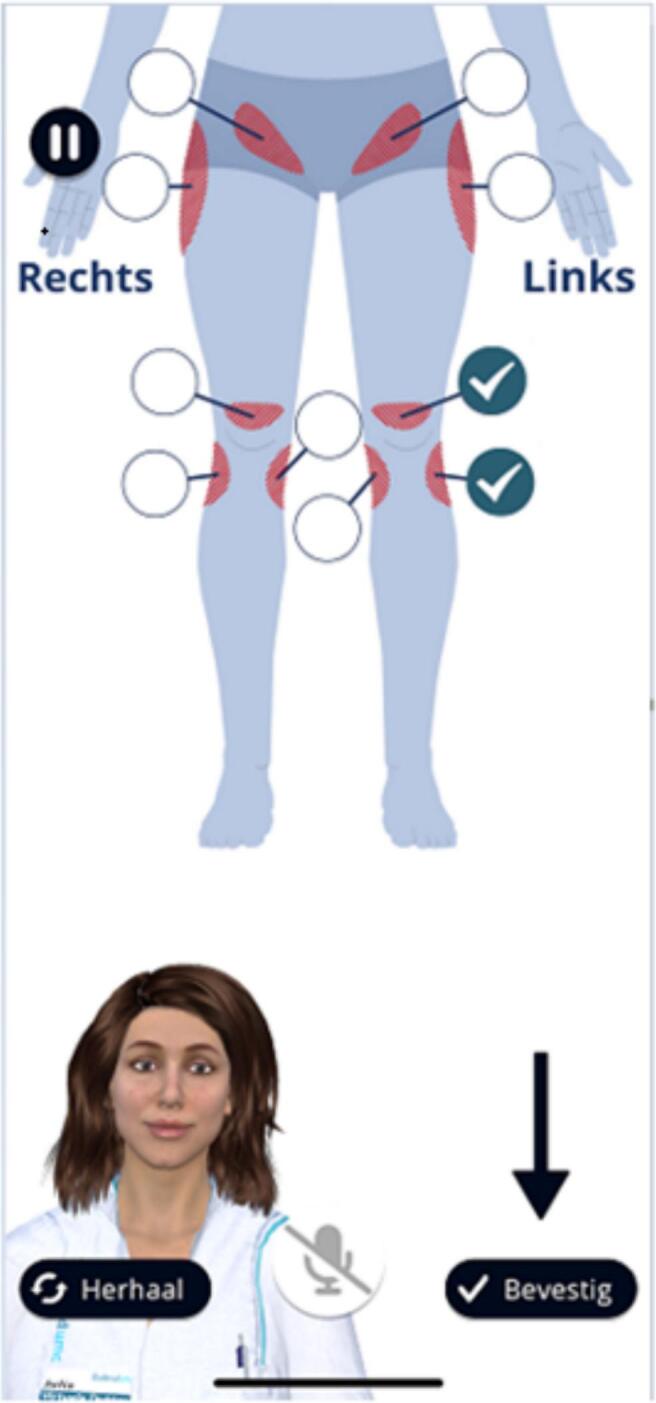
Screenshot of pointing out where the pain is located

After providing written informed consent, patients started a conversation with the VA using a hospital-provided mobile phone which had the mobile VA application pre-installed. Patients started the VA by pressing the “start” button on the start screen of the VA app on a mobile phone. Using speech, the VA introduced herself as “Julia, the digital assistant” and explained that she “was of course not a real doctor” but that she would help the real doctor by collecting relevant information prior to the in-hospital visit, which would be passed on to the real doctor. Next, she explained how to use the appropriate buttons to communicate (Fig. 1), which was practiced with a non-medical question. Location and severity of symptoms were asked first (Fig. 2), followed by questions on limitations with activities in daily life, including work and relationships. At the end, the system uses a Large Language Model (ChatGPT 3.5) to generate a summary of the collected information. The VA uses speech (ElevenLabs) to share this summary with the patient, after which patients were asked to add more detail and to correct any mistakes if necessary. After the session, the final version of the summary becomes available to the (human) healthcare professionals as a note in the EHR. The history taking questions and treatment information were provided by a consultant orthopedic surgeon (SvdG), experienced in treating patients with OA.

The VA was developed by Radboud University Medical Centre (RUMC, Nijmegen, the Netherlands) in corporation with a private company (The Simulation Crew, Nijmegen, the Netherlands). The VA, designed with the appearance of a female medical staff member of RUMC, was first tested with the help of Radboud UMC employees. Based on their feedback a second version was developed to facilitate a natural, open conversation between a patient and the VA. The second version was tested with low literate volunteers (*n* = 5). Their feedback was used to further modify the VA. The resulting third version was used in this study. The VA responds empathetically and asks relevant follow-up questions based on patient input. Using speech recognition (Google Dialogflow), a rule-based model guides the virtual assistant responses and conversation flow. This is complemented by an animation system that controls non-verbal behavior and lip synchronization (the SimulationCrew).

### 2.4 Measures

To measure feasibility and acceptability the 18-item Dutch modified version of the Artificial-Social-Agent Questionnaire (ASAQ), measuring 7 dimensions of VA was used. The Dutch version of the ASAQ was a translation from the original English questionnaire through three rounds of committee translations with 240 participants rating the English items and their translations [11,12]. Dimensions covered were (1) human-like behavior, (2) ease of use, (3) avatar appearance (4) avatar thrust worthiness, (5) how captivating is the conversation, (6) avatar thoughtfulness and (7) likelihood of future use.

A member of the research team supervised the conversation to make notes about the patients' behavior, the VA's behavior, and VA application usability, without intervening or providing (technical) assistance during the patient-VA conversation. After the conversation with the VA, patients were asked to complete the ASAQ and if they had any general remarks on their VA conversation experience. The supervising member of the research team also recorded his/her observations regarding the conversation of the patient with the VA and on the summary generated by the VA, including the number of digital hallucinations and how often information was missing. Any inaccurate or misleading statement generated by ChatGPT was deemed to be a digital hallucination [13]. The number of issues brought forward by the patient during conversation with the VA that were omitted in the summary was counted.

### 2.5 Analysis

Feedback from patients and supervisors from the research team was entered in a password protected spreadsheet, only accessible to the involved researchers documented on the delegation log. All completed questionnaires were digitalized and stored on a password protected and centralized hospital storage facility. Patient identifiers were replaced by a unique study identification number. Descriptive statistics were used to present demographics variables and questionnaire results, with categorical variables presented as numbers and proportions.

### 2.6 Ethical considerations

All eligible participants willing to participate provided written informed consent before study participation. This study was, according to Dutch law, considered exempt from full review by the regional medical ethical review board (N23.090).

## 3 Results

Forty-six patients were invited to participate, of which 40 (26 females, 14 male) agreed and were included. Reasons for not participating were lack of time (*n* = 4) or not interested (*n* = 2). Median patient age was 67 years (minimum: 47.3, maximum 87.5), 22 suffered from hip OA, 18 from knee OA. Overall, patients found history taking with a VA acceptable. The majority (36/40, 87.5 %) found the VA to behave like a human during the history taking conversation, 33/40 patients (82.5 %) found the VA easy to use, 31/40 patients (77.5 %) stated that they would consider to use the VA in the future again and 26/40 patients (65 %) found the conversation with the VA captivating. See Table 1 for detailed scores.

**Table 1:** Modified ASAQ scores. Values are percentages.

	Strongly disagree	Disagree	Neutral	Agree	Strongly agree
Humanlike behavior					
VA behaves like a human	0	5	2.5	80	7.5
VA reacts naturally	0	12.5	7.5	67.5	7.5
Conversation with VA is natural	0	5	15	72.5	2.5

Appearance					
VA has a pleasant appearance	0	2.5	7.5	75	10
VA has a fitting appearance	0	0	10	77.5	7.5

Easy to use					
Learning to communicate with VA is fast	0	7.5	5	70	12.5
VA is easy to use	0	10	7.5	62.5	15
	Trust				
I trust the VA	2.5	10	20	57.5	5
The VA understands me	2.5	10	12.5	65	7.5
The VA has no idea what she was doing	17.5	57.5	7.5	12.5	0

Captivating					
The VA conversation was captivating	2.5	7.5	22.5	55	5
I like the VA	2.5	0	12.5	65	15
The VA is boring	7.5	70	12.5	5	2.5

Thoughtful					
The VA was thoughtful	0	0	12.5	77.5	5
VA reactions fitted my feelings	0	12.5	22.5	57.5	5
It is unpleasant to deal with the VA	12.5	75	2.5	5	0

Future use					
I will use the VA again in the future	2.5	12.5	7.5	62.5	7.5
I would encourage others to use the VA	2,5	0	7.5	77.5	5

VA: Virtual assistant; ASAQ: Artificial Social Agent Questionnaire.

Twenty-two digital hallucinations occurred in the summary of 16 patients. The number of hallucinations per summary varied from 1 to 4. Hallucinations varied from reporting the absence of a variety of cardiovascular symptoms, which were not discussed during the interview, to the statement that a patient supported his knee for comfort, which was also not mentioned in the interview.

Time to complete the patient-VA interview ranged from 8.6 min to 17.4 min (median: 10.8 min). Multiple patient suggestions for improvement of the VA usability were recorded, for example increasing the size of the speech button, adding a function to go backwards in the VA conversation and adding questions on previous hip or knee OA treatment.

## 4 Discussion and conclusion

This study explored the feasibility and acceptability of using an avatar for history taking with older adults seeking orthopedic care for osteoarthritis. We demonstrated that the majority of patients thought the conversation with the tested VA to be acceptable. With the patient feedback on the tested prototype, VA appearance and behavior can be improved further to achieve an even higher rate of acceptance. Furthermore, avatars can of course also be designed to support other healthcare professionals or other phases of the patient care process in orthopedic surgery, thereby holding the promise to relieve the pressure on the current healthcare system. This interest is mirrored in similar projects undertaken in other medical specialties [14]. In addition, VA's might be used not only to gather information, but also to support patients in providing them with information [15].

Acceptance by patients is of course the prerequisite for implementation of digital care concepts such as the one studied in this paper. For clinical implementation, it is of great importance that the summary derived from the AV-led consultation is without missing relevant details and without errors due to hallucinations. In our study these hallucinations were easily identified but their occurrence requires further development and improvement of the AI-model.

Our findings are in concordance with previous literature that showed that VA software is feasible to implement and acceptable to patients and staff in a health care setting [16] and that technology is well accepted by older patients [17]. A previous systematic review included 17 studies reporting on 14 different conversational agents, of which 3 were for conducting clinical interviews, all with diagnostic purposes in mental health and sleep disorders [18]. User experience satisfaction was overall high but many issues in spoken language and incomplete and inconsistent responses were recorded. These conversational issues were much less present in our study, demonstrating the exponential developments in recent years in AI and LLMs particularly.

Besides further fine-tuning of VA functionality, future developments might be aimed at expanding the depth of questions based on the participants' responses. These are easily overlooked in regular face-to-face history taking but are known to have substantial effect on outcome of orthopedic treatments, including surgery [19,20]. Another challenge is to retrieve data from these patient-VA conversations on which AI applications can predict diagnosis and treatment options. Machine learning algorithms have proven to be capable for this purpose based on information collected with digitalized questionnaires [21,22]. Digitalized questionnaires, which are easy to apply for CAHT, have inherent limitations compared to normal speech. Answering options are usually in Likert scale, Visual Analogue Slider or Numeric Rating Scale format, reducing the extent and depth of the information the patient is asked to provide while on the other hand providing more opportunities to feed into prediction models. Illiterate patients or those who have severe difficulty with reading are also at a disadvantage or even unable to use such digitalized questionnaires.

Trust, knowledge, regard, and loyalty are the cornerstones that form the doctor-patient relationship, which is considered a cornerstone of health care [23]. Interestingly, in our study 62.5 % of the patients trusted the VA, which can be considered a reasonable score for a prototype VA and not so deviant from a study on patient trust in their human physician by Kao et al. They reported that 69.4 % of patients completely trusted their physician, 19.8 % mostly trusted and 8.0 % somewhat trusted their physician. Patient trust was related to having a choice of physicians, having a longer relationship with their physician, and trusting their managed care organization [24]. It is unknown if these factors are also relevant for trust in digital health care assistants.

Using CAHT, a physician can prepare the consultation based on information provided by the patient on his or her disease status, symptoms, limitations and reason to seek medical help. This can lead to more personalized face-to-face consultations in which the patient recognizes that the physician is prepared and informed about the patients' complaints and medical needs. This most likely contributes to building trust into the patient-doctor relationships.

Virtual assistants might also be applied for other health care conversations than history taking. For example, standard follow up moments after treatment or when the patient is contacting the hospital for questions or appointment scheduling. This will require development of more general AI algorithms but holds the promise of reducing the current strain on health care.

Using virtual assistants introduce different forms of risk. Omissions of relevant data or digital hallucinations negatively affect the quality of a medical history summary produced by a VA. History taking data collected by a VA might also contain signs that warrant immediate medical attention, while there is likely delay in the presentation of this data to a health care professional. The ethical and legal implications of these issues are not yet fully matured. Also the processing, storage and further analysis of the acquired data is subject to (inter)national laws which rightfully protect the individual patient but impose a significant (financial) burden on VA development.

### 4.1 Limitations

Since this was a feasibility and acceptability study, no a priori sample size calculation was conducted. With all included patients recruited in a single hospital, our patient sample might not fully represent patient characteristics from other hospitals or health care institutions. Our convenience sampling recruitment approach might have resulted in an overrepresentation of patients who favor new technologies, although only a small number (*n* = 6) declined participation. Furthermore, only patients suffering from hip or knee OA were eligible, limiting validity for other orthopedic complaints. We also used a pre-installed VA app, and patients might have difficulty accessing and installing apps on their mobile devices.

In our study, feasibility and acceptability of the VA were tested after the patient had consulted an orthopedic surgeon and with an observer present during the patient-VA conversation, which might introduce a bias towards favorable reporting by the patient. Since our study showed that VA use in a controlled setting is feasible and that patients accept this technology, future research should include how well patients would manage the use of a VA while they are at home without access to a pre-installed app. Furthermore, it should explore how health care professionals view the use of a VA in their practice, if this form of CAHT contributes to more detailed face-to-face history taking and how answers given with natural speech can be used in AI models that support diagnostic and treatment decisions. However, considering this was a feasibility and acceptability study, we were able to include quite a large group of patients who had a representative age and gender distribution for patients suffering knee or hip OA [25].

### 4.2 Innovation

Although previous investigators have described the use of digital conversational agents in health care, none were in the field of orthopedic surgery for osteoartritis, a common condition in older patients. In this study, we describe the use of a digital conversational agent specifically designed for patients with hip or knee osteoartritis. Our study results challenges the widespread belief that older patients are unwilling or unable to use digital conversational agents in health care and encourages further development of such agents for patients with hip or knee complaints due to osteoartritis.

### 4.3 Conclusion

Older patients with hip or knee osteoartritis found the VA conversation easy to use, captivating and would consider using this technology again. Future research should study the accuracy of an AI-generated summary and explore feasibility of avatar use at home before hospital consultation.

## CRediT authorship contribution statement

SG, TT, KS and WvdW contributed to the study design; WvdW and MJ collected the data in collaboration with TT; SG, TT and WvdW planned the statistical analysis, later performed by WvdW; all authors contributed to interpreting the results and revising the manuscript.

## Declaration of competing interest

The authors declare that they have no known competing financial interests or personal relationships that could have appeared to influence the work reported in this paper.

## Appendix A Supplementary data

Modified ASAQ questionnaire. Image 1

Dutch modified ASAQ results from low literate volunteers (n=5). Image 2
