# Supplementary material for: Human interaction with a virtual assistant in preparation for in-hospital orthopedic consultation. A feasibility and acceptability study in older adults with osteoarthritis
Source: PEC Innov. 2025 Nov 19;8:100446. doi: 10.1016/j.pecinn.2025.100446 (PMC12702030; doi:10.1016/j.pecinn.2025.100446)
Supplement: Supplementary file 1 — Modified ASAQ questionnaire. [file mmc1.pdf]

Supplement 1. Modified ASAQ questionnaire (English and Dutch versions)

1. The virtual assistant behaves like a human.

Strongly disagree	disagree	Neutral	Agree	Strongly agree
o	o	o	o	o

2. The virtual assistant looks pleasantly.

Strongly disagree	disagree	Neutral	Agree	Strongly agree
o	o	o	o	o

3. The virtual assistant reacts in a natural manner.

Strongly disagree	disagree	Neutral	Agree	Strongly agree
o	o	o	o	o

4. The appearance of virtual assistant is fitting.

Strongly disagree	disagree	Neutral	Agree	Strongly agree
o	o	o	o	o

5. The virtual assistant is easy to use.

Strongly disagree	disagree	Neutral	Agree	Strongly agree
o	o	o	o	o

6. Learning how to communicate with the virtual assistant goes fast.

Strongly disagree	disagree	Neutral	Agree	Strongly agree
o	o	o	o	o

7. The conversation with the virtual assistant goes in a natural manner.

Strongly disagree	disagree	Neutral	Agree	Strongly agree
o	o	o	o	o

8. I like the virtual assistant.

Strongly disagree	disagree	Neutral	Agree	Strongly agree
o	o	o	o	o

9. I will use the virtual assistant in the future again.

Strongly disagree	disagree	Neutral	Agree	Strongly agree
o	o	o	o	o

10. The virtual assistant is boring.

Strongly disagree	disagree	Neutral	Agree	Strongly agree
o	o	o	o	o

11. The virtual assistant is not nice to deal with.

Strongly disagree	disagree	Neutral	Agree	Strongly agree
o	o	o	o	o

12. The conversation with the virtual assistant is captivating.

Strongly disagree	disagree	Neutral	Agree	Strongly agree
o	o	o	o	o

13. I trust the virtual assistant.

Strongly disagree	disagree	Neutral	Agree	Strongly agree
o	o	o	o	o

14. The virtual assistant is understands me.

Strongly disagree	disagree	Neutral	Agree	Strongly agree
o	o	o	o	o

15. The virtual assistant is thoughtful.

Strongly disagree	disagree	Neutral	Agree	Strongly agree
o	o	o	o	o

16. The reactions of the virtual assistant fit how I feel.

Strongly disagree	disagree	Neutral	Agree	Strongly agree
o	o	o	o	o

17. The virtual assistant has no idea what she is doing.

Strongly disagree	disagree	Neutral	Agree	Strongly agree
o	o	o	o	o

18. I would encourage others to use the virtual assistant

Strongly disagree	disagree	Neutral	Agree	Strongly agree
o	o	o	o	o
