# Supplementary material for: Human interaction with a virtual assistant in preparation for in-hospital orthopedic consultation. A feasibility and acceptability study in older adults with osteoarthritis
Source: PEC Innov. 2025 Nov 19;8:100446. doi: 10.1016/j.pecinn.2025.100446 (PMC12702030; doi:10.1016/j.pecinn.2025.100446)
Supplement: Supplementary file 2 — Dutch modified ASAQ results from low literate volunteers (n=5). [file mmc2.pdf]

Supplement 2. Dutch modified ASAQ results from the low literate volunteers (n=5).

Modified ASAQ scores. Values are numbers

	Strongly disagree	Disagree	Neutral	Agree	Strongly agree
Humanlike behavior					
VA behaves like a human	0	0	0	4	1
VA reacts naturally	0	0	0	4	1
Conversation with VA is natural	0	0	0	2	3
Appearance					
VA has a pleasant appearance	0	0	0	3	2
VA has a fitting appearance	0	0	0	5	0
Easy to use					
Learning to communicate with VA is fast	0	0	1	2	2
VA is easy to use	0	0	1	2	2
Trust					
I trust the VA	0	0	0	4	1
The VA understands me	0	0	0	2	3
The VA has no idea what she was doing	3	2	0	0	0
Captivating					
The VA conversation was captivating	0	0	0	3	2
I like the VA	0	0	0	2	3
The VA is boring	2	3	0	0	0

Thoughtful					
The VA was thoughtful	0	0	0	3	2
VA reactions fitted my feelings	0	0	0	3	2
It is unpleasant to deal with the VA	1	2	0	2	0
Future use					
I will use the VA again in the future	0	1	0	2	2
I would encourage others to use the VA	0	0	0	2	3

VA: Virtual assistant; ASAQ: Artificial Social Agent Questionnaire
